# Supplementary figures and images for: The Role of the Gut Microbiota in the Effects of Early-Life Stress and Dietary Fatty Acids on Later-Life Central and Metabolic Outcomes in Mice
Source: mSystems. 2022 Jun 13;7(3):e00180-22. doi: 10.1128/msystems.00180-22 (PMC9238388; doi:10.1128/msystems.00180-22)

A.

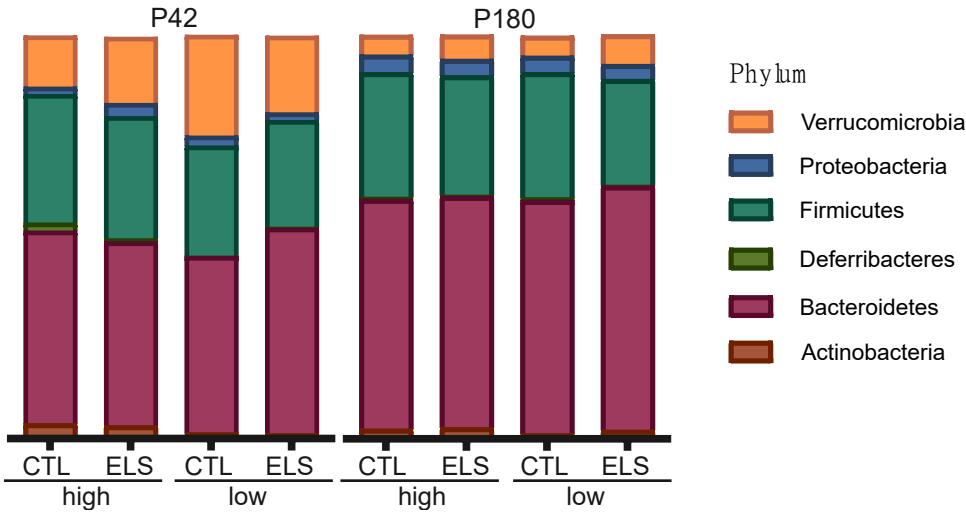

B.

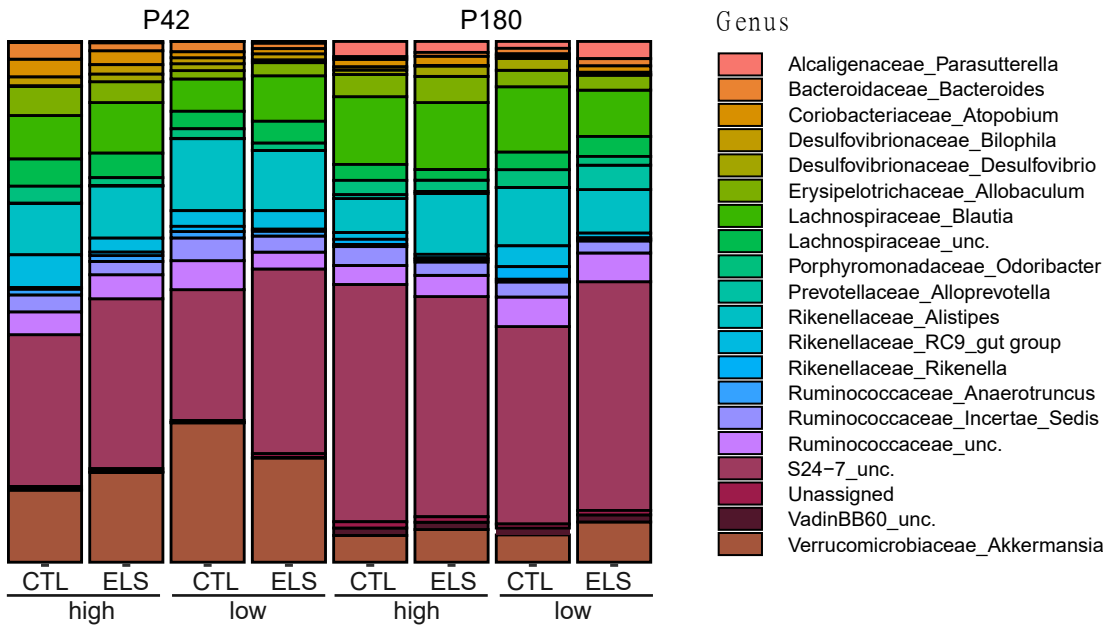

Supplement: FIG S1 [file msystems.00180-22-sf001.pdf]

A. Erythrocytes fatty acids

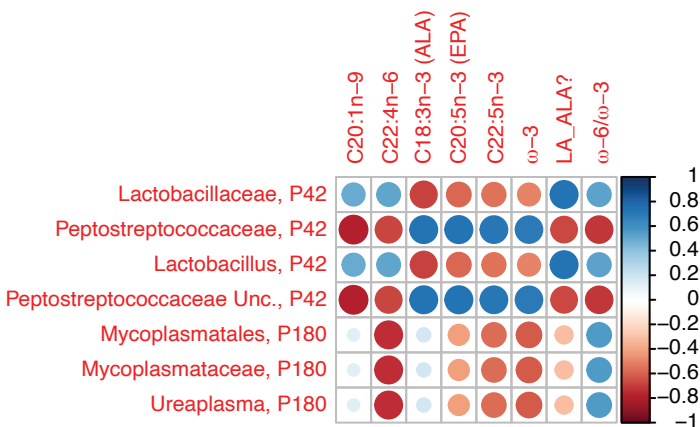

B. Liver fatty acids

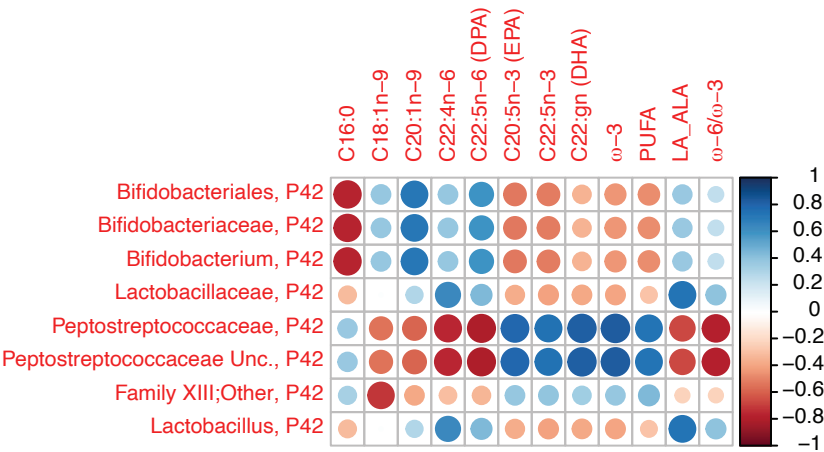

Supplement: FIG S2 [file msystems.00180-22-sf002.pdf]
