# Supplementary material for: The Role of the Gut Microbiota in the Effects of Early-Life Stress and Dietary Fatty Acids on Later-Life Central and Metabolic Outcomes in Mice
Source: mSystems. 2022 Jun 13;7(3):e00180-22. doi: 10.1128/msystems.00180-22 (PMC9238388; doi:10.1128/msystems.00180-22)
Supplement: TABLE S2 [file msystems.00180-22-st002.pdf]

Table S2. Microbiota (P42) at several taxonomic levels correlated with P42 metabolic outcomes. Correlations with 0.7 < *Rho* < - 0.7 are displayed.

	Taxonomic level	Body weight	Leptin levels	Inguinal fat	∑ White fat
Bacteroidetes	Phylum			-0,77	
Bacteroidales	Order			-0,77	
Porphyromonadaceae	Family	0,73			
Odoribacter	Genus	0,73			
S24-7	Family				-0,72
S24-7 Uncultured Bacterium	Genus				-0,72
S24-7 Ambiguous_taxa	Genus		-0,83		
Proteobacteria	Phylum				
Enterobacteriales	Order		0,71		
Enterobacteriaceae	Family		0,71		
Escherichia-Shigella	Genus		0,71		
Firmicutes	Phylum				
Clostridiaceae 1	Family		0,85		
Clostridium sensu stricto 1	Genus		0,85		
Marvinbryantia	Genus		0,78		
Christensenella	Genus		-0,71		
