# Supplementary material for: The Role of the Gut Microbiota in the Effects of Early-Life Stress and Dietary Fatty Acids on Later-Life Central and Metabolic Outcomes in Mice
Source: mSystems. 2022 Jun 13;7(3):e00180-22. doi: 10.1128/msystems.00180-22 (PMC9238388; doi:10.1128/msystems.00180-22)
Supplement: TABLE S1 [file msystems.00180-22-st001.pdf]

Table S1. Composition of experimental high and low ω-6/ω-3 PUFA diets (grams/kilogram diet). Abbreviations: PUFA: polyunsaturated fatty acids, MUFA: monounsaturated fatty acids, LA: linoleic acid, ALA: α-linolenic acid, SFA, short chain fatty acids

**Ingredient**	**High ω-6/ω-3**	**Low ω-6/ω-3**
Cornstarch, pregelatinized	397.5	397.5
Casein	200.0	200.0
Maltodextrin 10 DE	132.0	132.0
Sucrose	100.0	100.0
Cellulose	50.0	50.0
Mineral premix	35.0	35.0
Vitamin premix	10.0	10.0
L-cystein	3.0	3.0
Choline CL (50%)	2.5	2.5
Oil blend	70.0	70.0
Coconut oil. hydrogenated	23.8	23.6
Peanut oil	21.3	20.0
Safflower oil	20.2	6.7
Linseed oil	2.2	19.7
Soybean oil	2.5	_
Fatty acids (% total fatty acids)		
C6:0	0.1	0.1
C8:0	1.9	1.9
C10:0	1.9	1.9
C12:0	15.3	15.4
C14:0	6.9	6.9
C16:0	9.6	9.1
C18:0	6.0	6.3
C20:0	1.0	0.9
∑ SFA	42.6	42.4
C18:1	21.6	22.1
C20:1	0.6	0.4
∑ MUFA	22.1	22.6
C18:2n-6 (LA)	30.6	17.3
C18:3n-3 (ALA)	2.0	15.3
∑ PUFA	32.6	32.6
LA/ALA	15.3	1.1
